# An Inquiry into the Nature and Causes of Epilepsy; with the Function of the Spleen and the Use of the Thyroid Body, &c. &c.

**Published:** 1843-04

**Authors:** 


### Art. XIII.

*An Inquiry into the Nature and Causes of Epilepsy; with the Function of the Spleen, and the use of the Thyroid Body, &c. &c.* By John Jackson, Member of the Royal College of Surgeons.—*London*, 1842. 8vo, pp. 107.

A maiden essay to explain several difficulties by which the wisest physiologists of all ages have confessed themselves foiled, indicates a man who has no ordinary confidence in his own power; and that our author is not, in self-distrust, one of the crowd, is well proved by the spirit in which he writes. It is a style not of question or suggestion, but of downright dogmatic assertion, in which he lays down the following doctrines:—Ligature of the superior cava produces cerebral congestion and insensibility; ligature of the same vein, below the entrance of the vena azygos, produces cerebro-spinal congestion, and thence both insensibility and convulsions. Insensibility and convulsions in general, and epilepsy in particular, are therefore due to cerebro-spinal congestion. This congestion can be produced only by obstruction in the lower part of the vena cava superior; and such an obstruction can be due to nothing but an excessive force of the current through the inferior cava, making a rush of blood into the right auricle; and this rush is due to an excessive contraction of the spleen forcing the blood, in a sort of torrent, through the venous system of the liver.

The foundation of the greater part of the book, then, rests on the assertion that it is the office of the spleen, as an assistant organ of the circulation, to force blood through the liver. "It is the spleen which propels the portal blood through the portal trunk, branches, plexuses, and hepatic veins, to the inferior cava and auricle, and regulates the quantity and force with which it is propelled." (p. 27.)

It will probably content our readers if we let them know the style and value of the work, by examining each of the grounds on which this new account of the office of the spleen is founded.

It is said,—"The *vis a tergo* of the heart's action is not adequate to propel the blood through the venous and two capillary systems, and an additional power is necessarily required" to propel it through the liver. Now no proof of this is adduced, but it *has* been proved that, after death, a syringe worked with no greater force than the hæmadynamometer indicates as the force of the left ventricle, *is* capable of propelling fluid through the liver, and that without the sucking influence of inspiration. The general and fundamental assertion is therefore at once, and in general, disproved.

The grounds in particular on which it is made, are that there are peculiar difficulties in the passage of the blood through the abdominal vessels. Let us examine them. First, these vessels are said to be very tortuous. We were not aware of the fact. With the exception of the splenic artery and a small part of the coronaria ventriculi, the intra-abdominal arteries and veins run as straight as those of most parts of the body. Secondly, these vessels anastomose very often. But, the meeting of currents presents no hinderance to the circulation when there are sufficient side-channels for the blood to pass through; and of these there are many in all this part of the vascular system. But thirdly, says Mr. Jackson, the ascent of the blood through the roots of the portal vein is not aided by muscular contraction, nor is its descent prevented by valves. True: but there is here, as we shall presently show, a grievous defect in his physiology.

He institutes a comparison between the arteries of the lower extremities and those of the digestive organs, and he says that the contrast between them is very strong, especially in regard to the absence of anastomoses and of tortuosity in the former. The tortuosity, as we have said, is equally slight in both; and, for the anastomosis, there is no contrast, but a similarity. Mr. Jackson surely forgot the existence of plantar and dorsal arches, of anastomosing arteries, the junctions between the anterior fibular and anterior tibial, and between all the arteries of the leg around the ancle-joint. In proportion to the number and size of its arteries, there are unquestionably as many anastomoses in the leg as in the intestines or mesentery. But there is a contrast, it is maintained, in the matters of muscular pressure and of valves; and this is supposed to be the more important, because the blood has to move upwards.

This terrible influence of gravity is one of the most important items in Mr. Jackson's assertions: but he both misunderstands and misuses the facts of the case. He loses sight of the fact, that there is as high a column of blood in the arteries as in the veins below the heart, and that, therefore, the blood in both those sets of vessels is supported at an exact equilibrium, so that, when the two columns are each and together continuous, and both sets of vessels are healthy, the blood can be moved, where there are no valves, with equal facility in either direction. He deems the occurrence of varicose veins of the legs important, as indicating that although the same condition does not occur within the abdomen, yet there must be great hinderance to the blood. An ordinary man would have supposed, in the absence of a similar consequence, that a similar precedent condition does not exist; but our author can overlook the facts that the column of blood is more than twice as high, and therefore the pressure more than twice as great, upon the veins of the leg, as upon those of the digestive canal; and that varicose veins are the cause, not the effect, of difficulty in the circulation, being produced solely by the statical pressure of the blood, without any reference to its motion.

As to the valves, their use is also entirely mistaken. They have, it is well known, no relation to the gravitation of the blood. The venous column, as we have said, is supported by the arterial; and even when the blood is at rest, and the body erect, the valves cannot be forced down so long as the columns in the veins and arteries are continuous, for the pressure must be the same on both sides of them. Besides, suppose a pair of valves *could* be forced down by the weight of the blood above them: the case of the blood below them would be as bad as before; for what matters it whether its onward course be prevented by a superincumbent column of blood, or by a pair of valves closed over it? for, unless the valves be completely closed, the pressure from above is not diminished, and when once closed they cannot be opened unless the blood below them can force on that which is above them. The valves, then, we repeat, have nothing to do with the gravitation of the blood: they are found in veins in which the blood moves downwards, as well as in those in which it moves upwards; in short, in all the parts in which the veins are subject to muscular or any other habitual local external pressure, and in all of these their purpose is, to make as much as possible of that pressure efficient to the propulsion of the blood towards the heart, in a manner which Mr. Jackson might have learnt from any good system of physiology. Now, the veins of the digestive organs are not subject to this kind of pressure: valves, therefore, in them would be useless, and they do not exist. According to our author's view, their absence would be a great omission on the part of the Creator.

From the midst of all this error, Mr. Jackson has omitted three facts, which, though they are directly opposed to his views, are more important and exact than any he has inserted: viz., that in the splenic vein itself, on which the spleen must act most directly, the blood moves horizontally, and therefore *not* against gravitation; that through the liver, in which alone, after the portal trunk, the action of the spleen is exerted, the ascent in man is not more than an inch or two; and that, in this respect, the relation between man and quadrupeds is the very reverse of that on which he founds one of his chief arguments for his notion of the spleen's office, for the blood has to make a greater ascent in the hepatic veins of the latter than in those of the former.

We look in vain for one fact, or one correct argument, to prove that there is a peculiar difficulty in the passage of the blood through the digestive organs, or a necessity for any extra-propelling force; and we think we shall long look, with as little success, for any author making more mistakes in his first thirty pages than Mr. Jackson has made. But we come now to a more extraordinary assertion than any yet reviewed:

"The spleen, by its structure, properties, and position, and by its connexion with the trunk of the portal system by the splenic vein, is eminently adapted for the function of propelling the blood through the portal trunk, branches, plexuses, and hepatic veins." (p. 28.)

The sum of the facts of structure, &c., by which the spleen is thus eminently adapted to be a second heart, is that its fibrous coat and septa are elastic, and that there is some suspicion (and only a suspicion,) that they have a peculiar vital contractility, so that independently of its elasticity, the spleen may be able occasionally to discharge some of its blood. But Mr. Jackson can afford to forego this little, and, as we admit, very unimportant evidence for his notions: for his *proof* that the spleen is a heart is established by its analogy to—the placenta!

The points of analogy are:—1st. That "the circumference of both is more or less lobulated or fissured;" on which ground the brain, the lungs, the fœtal kidneys, and the pancreas, must be closely analogous to the placenta, for they are all more lobulated than the spleen. 2d. That "the spleen is enveloped by an elastic capsule,—the placenta is inclosed between two layers of an elastic membrane, the decidua;"—between which there is about as much analogy as between the dura mater and the chorion, or between caoutchouc and tissue-paper. 3d. That "both organs present a spongy and reticulated texture, consisting almost wholly of vessels, and chiefly of veins;" a good example of the judiciously indefinite style in descriptions which are intended to establish an analogy between things widely different. 4th. That the nerves of both are small; the fact being that the nerves of the spleen, when compared with those of the other viscera of organic life, are *not* small, but rather large, and that those of the placenta are *so* small that they have never yet been seen by any credible anatomist. Lastly, both organs are possessed of a peculiarly contractile power. Reserving this last, which is perhaps the most absurd of all, did you ever, good reader, meet with such a set of analogies in your life?

With respect to this contractile power of the placenta, from which a similar power is, by analogy, deduced for the spleen, Mr. Jackson's argument is, as usual, an assertion. He says, naïvely enough,—

"Whether or not it be conceded that the spleen is the organ which subsequently to birth propels the blood through the portal plexuses and the hepatic veins to the inferior cava and right auricle......I presume there are none who will be disposed to deny that the placenta performs that function in the fœtus." (p. 34.)

Disposed to deny it? Why! is there another man living who would assert such a thing? Need we say that there is not even the suspicion of a fact in favour of such a notion? After this, and when we assure them that all the rest of the book is of the same kind as that which we have quoted, and some of it even more absurd, our readers will hardly expect us to go on disproving what Mr. Jackson has written. One passage, however, is too *good* to be omitted.

"Inspiration," he says, "is commonly supposed to have the effect of facilitating ingress into the auricle by the inferior as well as by the superior cava. It appears, however, not very comprehensible, indeed to carry with it a contradiction, that inspiration should facilitate the ingress of blood by *both* venæ cavæ; that the same act should increase the impetus of two conflicting currents; that it should cause *descent* of the one and *ascent* of the other." (p. 62.) .......... Perhaps it is simply by *gravitation* that *inspiration* increases the force of ingress into the auricle by the superior cava." (p. 87.)

Oh! that some good friend had either prevailed on the author not to write, or else had taught him that the pressure of the atmosphere, like that of all fluids, is in all directions—equal.

We regret to be obliged to expose so many errors in a few pages, but we hope, and from his style we are led to believe, that Mr. Jackson will be more instructed than vexed by our remarks. On the next occasion he had better choose an easier subject; and we will suggest to him this problem (founded on his errors), of which he may apply the solution to other questions in physiology:—If the blood *be* forced rapidly through the splenic vein, it must be materially retarded by each of the slowly-moving currents which join it from the inferior, and superior, mesenteric and other veins; and to each of these it has to impart some of its velocity, till they both move together at the same rate. How much of its extra force, then, will remain at the vena portæ? And, in general, what is the influence of slow currents of venous blood passing at various angles into those which are moving more rapidly? a condition which probably frequently exists, though not at the splenic vein. Or this:—When the spleen contracts (if it have a power of contracting in mass), what would be the difference (if any) between the retardation of the blood in the splenic artery, and its acceleration in the splenic vein?

